# OA04.03. Neural responses to the mechanical characteristics of a spinal manipulation: effect of varying direction of the applied thrust force

**DOI:** 10.1186/1472-6882-12-S1-O15

**Published:** 2012-06-12

**Authors:** J Pickar, W Reed, C Long, G Kawchuk

**Affiliations:** 1 Palmer Center for Chiropractic Research, Davenport, USA; 2 University of Alberta, Edmonton, Canada

## Purpose

A goal of our laboratory is to identify mechanisms of action operative during the body-based practice of spinal manipulation. Spinal manipulation can be identified by a number of mechanical characteristics including but not limited to thrust rate, magnitude, site, and direction. Because neural mechanisms are thought to contribute to its clinical effects, we studied spinal manipulation during a series of experiments to identify mechanical characteristics that affect responses from sensory neurons innervating paraspinal tissues. Presumably specific parameters related to these characteristics relate to successful clinical outcomes. In this study we determined the effect of manipulative thrust direction on neural activity of proprioceptive afferents from lumbar paraspinal muscles.

## Methods

In anesthetized cat preparations (n=18), a simulated spinal manipulation (100ms thrust duration) was delivered in each of 5 directions [posterior-to-anterior (0°), cranialward (15° & 30°), and medialward (15° & 30°)] to the prone, intact lower lumbar spine (L_6_-S_1_)using the L_6_ lamina as the contact site. Importantly, we increased thrust force as we deviated thrust direction from vertical in order to keep the force component that was perpendicularly-oriented to the contact site constant at 21.3N (55% of an average cat’s body weight of 3.95kg). During the manipulative thrust, electrophysiological recordings from individual muscle spindle afferents innervating the L_6_ multifidus and longissimus muscles were obtained from L_6_ dorsal rootlets exposed through an L_5_ laminectomy.

## Results

Muscle spindle discharge frequency during P-A-0° thrust increased 81.6 (89.1, 98.0; 95% CI), during cranialward-15° increased 101.4 (93.1, 109.6), during cranialward-30° increased 82.8 (90.3, 96.6), during medialward-15° increased 104.6 (93.4, 115.8), and during medialward-30° increased 87.6 (69.8, 105.5) impulses/sec.

## Conclusion

Our results suggest that for the same vertically-oriented force component, thrust direction had little effect on muscle spindle responses. These data provide neurophysiological evidence that only the component of the manipulative thrust normally-oriented to the contact site is transmitted to deeper tissues.

